# Mapping changes in the obesity stigma discourse through Obesity Canada: a content analysis

**DOI:** 10.3934/publichealth.2022004

**Published:** 2021-11-15

**Authors:** Sara FL Kirk, Mary Forhan, Joshua Yusuf, Ashly Chance, Kathleen Burke, Nicole Blinn, Stephanie Quirke, Ximena Ramos Salas, Angela Alberga, Shelly Russell-Mayhew

**Affiliations:** 1 Healthy Populations Institute, Dalhousie University, PO Box 150000, Halifax, B3H 4R2, Canada; 2 School of Health and Human Performance, Faculty of Health, Dalhousie University, PO Box 150000, Halifax, NS, B3H 4R2, Canada; 3 Faculty of Rehabilitation Medicine, Department of Occupational Therapy, Corbett Hall, University of Alberta, 11405 87 Avenue NW, Edmonton, AB, T6H 2G4, Canada; 4 Research and Policy Consultant, Rönnebergsvägen 116, 29891 Tollarp, Sweden; 5 Department of Health, Kinesiology and Applied Physiology, Concordia University, 7141 Sherbrooke Street West, Montreal, Quebec, H4B1R6 Canada; 6 Werklund School of Education, University of Calgary, 3280 Hospital Drive NW, Calgary, AB, T2N 4Z6, Canada

**Keywords:** obesity stigma, weight bias, content analysis, health research, policy

## Abstract

**Background:**

Stigmatization of persons living with obesity is an important public health issue. In 2015, Obesity Canada adopted person-first language in all internal documentation produced by the organization, and, from 2017, required all authors to use person-first language in abstract submissions to Obesity Canada hosted conferences. The impact of this intentional shift in strategic focus is not known. Therefore, the aim of this study was to conduct a content analysis of proceedings at conferences hosted by Obesity Canada to identify whether or how constructs related to weight bias and obesity stigma have changed over time.

**Methods:**

Of 1790 abstracts accepted to conferences between 2008–2019, we excluded 353 abstracts that featured animal or cellular models, leaving 1437 abstracts that were reviewed for the presence of five constructs of interest and if they changed over time: 1) use of person-first versus use of disease-first terminology, 2) incorporation of lived experience of obesity, 3) weight bias and stigma, 4) aggressive or alarmist framing and 5) obesity framed as a modifiable risk factor versus as a disease. We calculated and analyzed through linear regression: 1) the overall frequency of use of each construct over time as a proportion of the total number of abstracts reviewed, and 2) the ratio of abstracts where the construct appeared at least once based on the total number of abstracts.

**Results:**

We found a significant positive correlation between use of person-first language in abstracts and time (R2 = 0.51, p < 0.01 for frequency, R2 = 0.65, p < 0.05 for ratio) and a corresponding negative correlation for the use of disease-first terminology (R2 = 0.48, p = 0.01 for frequency, R2 = 0.75, p < 0.001 for ratio). There was a significant positive correlation between mentions of weight bias and time (R2 = 0.53 and 0.57, p < 0.01 for frequency and ratio respectively).

**Conclusion:**

Use of person-first language and attention to weight bias increased, while disease-first terminology decreased in accepted abstracts over the past 11 years since Obesity Canada began hosting conferences and particularly since more explicit actions for expectations to use person-first language were put in place in 2015 and 2017.

## 1. Introduction

Stigmatization of persons living with obesity, attributed to weight bias, is increasingly recognized as an important public health issue, but one that has received relatively little attention until recently [1]. Weight bias is comprised of negative attitudes and beliefs about a person based on body weight [2]. Misconceptions about the causes and consequences of obesity are perpetuated by beliefs that obesity is a direct result of a lack of self-control with regards to eating and exercise behaviors and that body weight reduction is directly associated with improved health and well-being [3]. Obesity stigma is typically defined as the manifestation of weight bias through harmful social stereotypes that are associated with people living with obesity, and that are widely held societally, including among health care providers [4]. Bias and stigma are associated with negative mental and physical health as reported by children and adults living with obesity [5]–[8]. Simply put, obesity stigma is now understood to be harmful to health but remains prevalent and intractable [9]. In October 2019, the annual report of Canada's Chief Public Health Officer focused on different forms of stigma, including obesity stigma, and called for health education institutions and professional associations to transform the practices, curricula, and professional competencies of health providers [10]. In March 2020, a joint international consensus statement for ending stigma of obesity was published, calling for academic institutions, professional organizations, media, public-health authorities, and governments to “encourage education about weight stigma to facilitate a new public narrative about obesity, coherent with modern scientific knowledge” [11].

A multi-level, systems approach is required to reframe the prevailing stigmatizing narratives associated with obesity. Examples of strategies to reduce weight bias and stigma include adopting person-first language or terminology (i.e., people with obesity) instead of disease-first terminology (i.e., obese people, as well as other negative terms that people with obesity reject) and incorporating lived experiences of people living with obesity to promote empathy (i.e., people living with obesity are included in the research team or are provided with the opportunity to engage in research, practice or policy around obesity prevention or management) [12]. In 2008, the Board of Directors of Obesity Canada (formerly the Canadian Obesity Network, which was founded in 2006) identified reducing weight bias and obesity stigma as one of the network's top strategic priorities [13]. To this end, Obesity Canada began working towards reducing weight bias and obesity stigma through research, education and action. Activities to reduce weight bias and obesity stigma became an explicit focus across all network activities; the organization established a Public Engagement Committee to incorporate and amplify the lived experiences of people with obesity and three summits on weight bias and stigma were held [12],[14]. In 2015, the use of person-first language became a requirement in all documentation released by the organization [15]. These actions were strategic to advance Obesity Canada's mission to improve the lives of Canadians living with obesity through research, education and advocacy. In an effort to reduce weight bias and obesity stigma in the content of poster and oral presentations at the Canadian Obesity Summits, the use of person first language was included as part of the abstract submission guidelines for conference delegates attending the 2017 Canadian Obesity Summit (although this was not strictly enforced). The following is the original statement regarding person first language that was included in the abstract guidelines and remains in place today: “...People-first language is the standard for respectfully addressing people with chronic disease, rather than labelling them by their illness. Because of the importance of reducing bias associated with obesity, authors should not use ‘obese’ as an adjective or noun to describe and individual or group of people....”. Links to resources and examples of person-first language were provided for abstract authors.

To date, the impact of this intentional shift in strategic focus to reduce weight bias and obesity stigma through the use of a person-first-language policy within Obesity Canada is unknown. The aim of this study was to conduct a qualitative content analysis of conference proceedings at Obesity Canada conferences, which comprised Canadian Obesity Summits (COS) and Canadian Obesity Student Meetings (COSM), to identify whether or how constructs that either promote or reduce weight bias and obesity stigma have changed over time in accepted abstracts, as a measure of whether or how biased terminology has changed. These two types of conferences are biennial conferences taking place alternating years. The COS are open to established researchers and trainees while the COSM are only open to trainees.

## 2. Materials and methods

We conducted a content analysis of conference proceedings for Obesity Canada's conferences from 2008–2019 to identify how constructs related to weight bias or stigma may have been incorporated within conference abstracts, as a measure of whether/how biased terminology may have changed over time. The unit of analysis for this study was abstracts (n = 1790) that were accepted for presentation at COS and COSM from 2008–2019. Abstracts accepted for the conferences underwent a two-step review process. First, abstracts were reviewed for significance and scientific merit by an independent scientific review committee and graded on a scale of 1 to 5. Comments from reviewers were encouraged during this review process with regards the quality of each abstract. Next, the top ranked abstracts were considered for specific conference sessions or themes by a smaller conference organizing committee. Lowest ranked abstracts were rejected, and the number of abstracts accepted varied according to the physical space available at different conference locations. The abstracts were reviewed to identify whether the use of constructs related to weight bias or stigma had changed over time. Because abstracts were accepted for presentation at conferences, they represent information that was available in the public domain and therefore ethical approval was not required. Only abstracts that featured human subjects were included (n = 1437). Abstracts which involved animal models or focused on cellular or genetic mechanisms were excluded (n = 353). A total of 1026 abstracts were from COS and 411 were from COSM. Abstracts were imported into Nvivo 12 for coding.

**Table 1.** Description of constructs of interest.

Construct	Positive	Negative	Why this is important
Person-first terminology vs disease-first terminology used	People/person with obesity; person living in a large(r) body	Obese people/person; Use of negative terms that persons with obesity reject (e.g., chubby, morbidly obese, fat).	The appropriate use of person-first terminology is a means of assessing how attitudes towards obesity may have shifted [15].
Lived experience incorporated	People with obesity are engaged in research/practice/policy. Persons with obesity are engaged in the conference/presentation and other knowledge translation activities.	No lived experience inclusion; assumptions made by researcher/clinician.	An indicator of weight bias is whether people with lived experiences are being engaged in research/education/policy etc. Engaging persons with obesity to reduce weight bias has been shown to have a positive effect (empathy). Engagement of persons affected by stigma is a strategy that has been used to reduce stigma in other communities (diabetes, mental illness, HIV/AIDS, LGTBQ+) [10].
Weight bias/stigma	Weight bias/stigma is mentioned or considered as an important issue to be addressed.	Weight bias/stigma is ignored or negatively expressed (weight bias/stigma inappropriately used as a way to motivate behavior change in individuals).	Implicit or explicit weight bias can be expressed in words or actions, e.g., by describing a person as an object (e.g., a 500 pounder) or through negative attributes (e.g., in relation to non-compliance, appearance or character traits). Stigma is the overt form of bias [10].
Alarmist terminology	Individuals living with obesity are strong, productive and valuable contributors to Canada. They deserve to be heard and demand to be included; decisions on treatment, care, prevention or policy must be person-centered.	Obesity is a burden to the healthcare system and society. People with obesity are less productive, spend more healthcare dollars; The use of war metaphors in abstracts.	The portrayal of obesity as a burden to society can lead to victim blaming and shaming; Persons with obesity are seen as a burden to society which contributes to weight bias/stigma [16],[17].
Obesity as a modifiable risk factor vs obesity as a disease	Obesity is a complex chronic disease that is the result of the interactions of genetic, metabolic, behavioral and environmental factors; Obesity prevention interventions move beyond healthy eating and physical activity.	Obesity is not a chronic disease in itself but a risk factor for other chronic diseases; Obesity is caused mainly by modifiable behaviors (healthy eating and exercise). Healthy eating and physical activity interventions framed as obesity prevention strategies.	The narrative that obesity is a self-inflicted behavioural choice drives weight bias attitudes and beliefs; Framing of obesity as a chronic disease can reduce weight bias [13],[18],[19].

Content analysis is a form of thematic analysis that analyzes written, verbal or visual communication messages as a means to quantify certain phenomena [18]. Content analysis as a methodology has been applied to identify a broad range of phenomena, such as the most prevalent topics featured at an aging conference [20], gender messaging in parenting magazines [21] and children's food advertising [22]. It has also been successfully used to assess weight bias and stigma in a study by Heuer et al, that identified stigmatizing images across a range of media outlets [16]. Content analysis can be inductive (themes emerging from the data during the analysis process) or deductive (data analyzed using pre-determined categories based on what the research is designed to achieve) [18]. In this context, we applied a deductive analytical approach to assess whether there was a change (increase or decrease) in the appearance of five constructs of relevance to weight bias/stigma reduction over time. These constructs were agreed a priori by the authors as reflective of biased language and were 1) the use of person-first versus disease-first terminology; 2) the incorporation of lived experience in the work being described in the abstract; 3) whether weight bias or stigma is mentioned or considered as an important issue to be addressed; 4) the use of alarmist terminology to describe people living with obesity, and 5) language around whether obesity is viewed as a modifiable risk factor or as a disease. Table 1 provides a more detailed description of the constructs of interest. Each abstract was reviewed independently by two authors or research assistants with oversight by authors SFLK and MF. Discrepancies in coding were resolved through discussion. We calculated: 1) the overall frequency of use of each construct over time as a proportion of the total number of abstracts reviewed, and 2) the ratio of abstracts in which the construct appeared at least once based on the total number of abstracts, which accounts for a construct appearing multiple times within an abstract. Linear regression analyses were performed using Microsoft Excel to identify any correlations between these two measures and conference year.

## 3. Results

**Figure 1.**
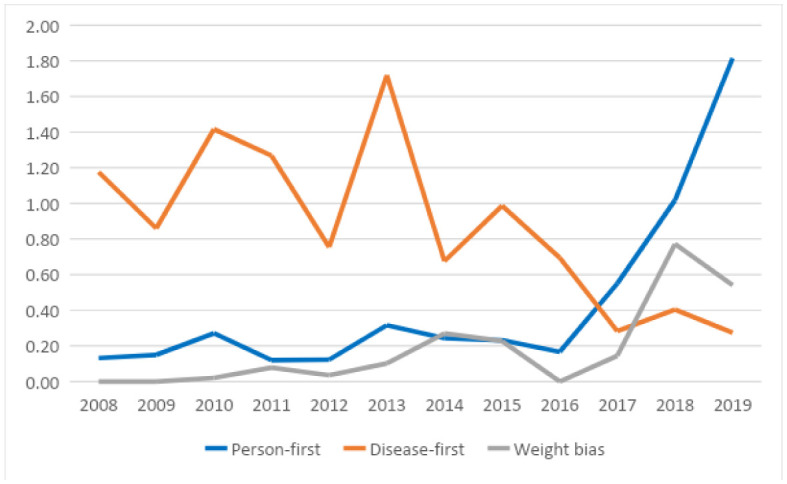
Frequency of use of person-first, disease-first and weight-bias constructs over time.

**Figure 2.**
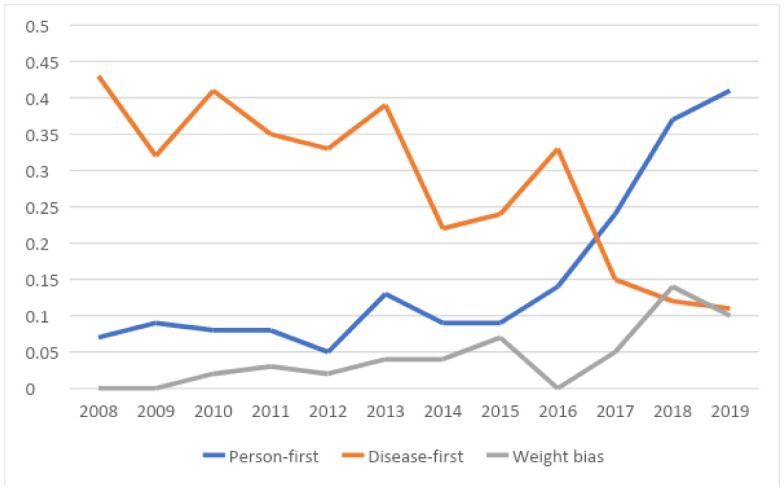
Ratio of person-first, disease-first and weight-bias constructs over time.

Table 2 shows the frequency and ratio of use of the relevant constructs in abstracts over time for all conferences, while Table 3 provides the ratio of abstracts where the construct appeared at least once relative to the total number of abstracts. From these it can be seen that there was a significant positive correlation between the use of person-first language in abstracts and time across both conference types and a corresponding negative correlation for the use of disease-first terminology. There was a significant positive correlation between mentions of weight bias and time. These significant effects remained when analyzed separately among COS abstracts, but only disease-first terminology remained significantly negatively correlated in the COSM abstracts. The frequency of abstracts that included or reported on lived experiences of obesity appeared to be low and did not vary over time in overall conference types or in sub-types (i.e., COS versus COSM). No significant trends were seen over time for use of alarmist terminology or framing of obesity as a disease or modifiable risk factor when considered as a ratio of the construct relative to the total number of abstracts. Figures 1 and 2 show the frequency and ratio of person-first, disease-first and weight bias constructs over time. Frequency of person-first and weight bias construct use increased significantly from 2015/2016 onwards, when person-first language became a requirement, initially in 2015 for all documents released by the organization and in 2017 when the requirement was applied to conference abstracts as a condition of acceptance.

**Table 2.** Frequency (ratio) of construct use over time (all conferences).

Year	Total no. of abstracts accepted (% of total)	Person-first terminology used	Disease-first terminology used	Lived experience incorporated	Weight-bias mentioned or considered	Alarmist terminology used	Obesity framed as disease	Obesity framed as a modifiable risk factor
2008	91 (na*)	6 (0.13)	39 (1.18)	0 (0.00)	0 (0.00)	7 (0.09)	0 (0.00)	11 (0.13)
2009	107 (83%)	10 (0.15)	34 (0.86)	3 (0.03)	0 (0.00)	14 (0.16)	4 (0.04)	10 (0.09)
2010	96 (89%)	8 (0.27)	39 (1.42)	1 (0.01)	2 (0.02)	5 (0.11)	4 (0.04)	13 (0.24)
2011	217 (79%)	17 (0.12)	75 (1.27)	6 (0.04)	6 (0.08)	19 (0.15)	6 (0.05)	28 (0.20)
2012	57 (90%)	3 (0.12)	19 (0.75)	0 (0.00)	1 (0.04)	2 (0.04)	0 (0.00)	1 (0.02)
2013	196 (80%)	25 (0.32)	76 (1.72)	3 (0.03)	7 (0.10)	13 (0.11)	2 (0.01)	15 (0.10)
2014	74 (95%)	7 (0.24)	16 (0.68)	0 (0.00)	3 (0.27)	3 (0.05)	1 (0.03)	9 (0.19)
2015	233 (90%)	21 (0.23)	56 (0.99)	13 (0.07)	16 (0.23)	19 (0.10)	8 (0.01)	18 (0.09)
2016	36 (na*)	5 (0.17)	12 (0.69)	0 (0.00)	0 (0.00)	0 (0.00)	11 (0.22)	0 (0.00)
2017	138 (96%)	33 (0.55)	21 (0.28)	4 (0.04)	7 (0.14)	1 (0.01)	0 (0.00)	2 (0.01)
2018	57 (93%)	21 (1.02)	7 (0.40)	3 (0.09)	8 (0.77)	8 (0.18)	8 (0.14)	11 (0.28)
2019	135 (70%)	56 (1.81)	15 (0.27)	4 0.05)	14 (0.14)	3 (0.02)	28 (0.21)	4 (0.05)
R^2^ value		0.51	0.48	0.31	0.53	0.16	0.35	0.04
P value		<0.01	=0.01	NS	<0.01	NS	<0.05	NS
F value		10.56	9.41	4.44	11.47	1.88	5.51	0.40
df		11	11	11	11	11	11	11

*Note: Acceptance rates were not available for these two years. In 2008, the review committee was organized by the host institution and in 2016, the conference was run in conjunction with the International Congress on Obesity (ICO) which coordinated abstract review for both ICO and COSM.

**Table 3.** Ratio of construct use over time (all conferences) as a proportion of all abstracts.

Year	Person-first terminology used	Disease-first terminology used	Lived experience incorporated	Weight-bias mentioned or considered	Alarmist terminology used	Obesity framed as disease	Obesity framed as a modifiable risk factor
2008	0.07	0.43	0	0	0.08	0	0.12
2009	0.09	0.32	0.03	0	0.13	0.04	0.09
2010	0.08	0.41	0.01	0.02	0.05	0.04	0.14
2011	0.08	0.35	0.03	0.03	0.09	0.03	0.13
2012	0.05	0.33	0	0.02	0.04	0	0.02
2013	0.13	0.39	0.02	0.04	0.07	0.01	0.08
2014	0.09	0.22	0	0.04	0.04	0.01	0.12
2015	0.09	0.24	0.06	0.07	0.08	0.03	0.08
2016	0.14	0.33	0	0	0	0	0
2017	0.24	0.15	0.03	0.05	0.01	0.04	0.01
2018	0.37	0.12	0.05	0.14	0.14	0	0.19
2019	0.41	0.11	0.03	0.1	0.02	0.09	0.03
R^2^ value	0.65	0.74	0.17	0.57	0.12	0.07	0.09
P value	<0.05	<0.001	NS	<0.01	NS	NS	NS
F value	18.73	28.37	2.00	13.30	1.34	0.77	1.01
df	11	11	11	11	11	11	11

## 4. Discussion

In this analysis, we sought to assess the impact of Obesity Canada's intentional shift in strategic focus to reduce weight bias and obesity stigma among members and the wider research and clinical practice communities. Our findings suggest that there has been a shift in the constructs of interest, which in turn points to a change in terminology around how people with obesity are described in research and clinical practice, along with increasing recognition of weight bias and stigma as constructs of relevance to the obesity discourse.

Although we cannot assume causation given the study design, we can see trends towards more frequent use of person-first language and less frequent use of disease-first terminology, as well as a trend towards more mentions of weight bias as an important construct for study in abstracts accepted over time, as illustrated in Figures 1 and 2. We did not see any significant trends over time in relation to the use of alarmist terminology or the incorporation of lived experiences as defined in Table 1. These constructs were also more difficult to assess. For example, it was difficult to analyze the incorporation of lived experiences through reading the abstracts alone as it was not always clear if individuals living with obesity were study participants, included as authors of the abstract or part of the presentation team, or if their lived experience was relevant to the study or presentation. It is therefore possible that lived experience was a feature of a presentation even though it was not mentioned in the abstract.

The increase in abstracts that incorporated person-first language from 2015 corresponds to the decision by Obesity Canada to require the use of person-first language in all documents released by the organization [15]. Although person-first language was a component of Obesity Canada's implicit, strategic focus from 2008, incorporating this explicitly into the abstract submission guidelines in 2017 appears to have led to increased compliance at the COS that year. It should be noted that in 2015, Obesity Canada also launched the Public Engagement Initiative which included the creation of a Public Engagement Committee composed of individuals living with obesity. In 2016, Obesity Canada's Board of Directors also decided to restructure the organization into a registered charity. The organization underwent a rebranding exercise, creating a new name (from Canadian Obesity Network to Obesity Canada), logo and mission statement which focused on improving the lives of Canadians affected by obesity. Since 2017, all of Obesity Canada's committees have added representation from people living with obesity. In 2018, Obesity Canada created a patient-centered obesity advocacy strategy, which has significantly increased the voices of people living with obesity in Canada. Furthermore, 2017 was also the year that the lived experiences of people living with obesity were more firmly embedded in the conference program as keynote talks starting off each day of the conference. Creating empathy has been identified as an important component to reduce weight-bias and stigma among health professionals [2].

Strengths of this study are the application of a robust methodology to identify constructs of relevance to weight bias and stigma reduction. Other strengths are the inclusion of all relevant abstracts that were accepted to the two national and student conferences over an 11-year time frame and the consensus-driven process for assessing abstracts as a means to identify the constructs of interest. In terms of limitations, there is likely a time lag with respect to the type of research that was submitted to the conferences, something that has been identified in the knowledge translation literature [23]. Research presented at any given conference reflects research funded and/or conducted in the preceding years, which may not be sensitive to shifts in policy. It would be anticipated that continued monitoring for constructs of interest over future conferences would help overcome these limitations. Further research is needed to see if such a change in approach translates to decreases in weight biased attitudes and beliefs among the public and health professionals. Additionally, our analysis was confined to accepted abstracts rather than all submitted abstracts. The acceptance rate for COS abstracts averaged 83% over the study period (range 70–96%), while the acceptance rate for COSM abstracts was slightly higher at 92% (range 89–95%), as might be expected for a student-focused conference (Dawn Hatanaka, personal communication). While we cannot be sure that submitted abstracts differed from accepted ones with respect to the constructs of interest, this represents a high acceptance rate.

Our findings suggest a need to more intentionally address weight bias and stigma through changing policy and practice and clearly communicating such changes [1]. Obesity Canada's strategic decision in 2008 provided an opportunity for such changes to take root. However, addressing obesity as a chronic disease and weight bias as a social determinant of health are two priorities that still need to be more firmly embedded in healthcare settings [14],[24],[25]. These changes take time to become adopted as standard practice and their adoption should continue to be monitored. For example, with the inclusion of a weight bias and stigma chapter in the 2020 Canadian Obesity Clinical Practice Guideline, Obesity Canada has reaffirmed the need to use person-first language in research, clinical practice and policy [26]. In 2018, the Canadian Obesity Advocacy Network, comprised of a group of diverse organizations, prioritized the need to reframe obesity as a chronic disease by having consistent messaging and using person-first language in organizational reports, messaging and education [27]. Some research has also shown that framing obesity as a disease is associated with lower weight bias in health professionals [28],[29] and the public [29]. In 2019, the Public Health Agency of Canada called on all Canadians to end multiple forms of stigma in our society, including obesity stigma [10].

## 5. Conclusions

In this content analysis of abstracts accepted to a leading Canadian obesity conference series, we found that the use of person-first terminology increased, and the use of disease-first terminology decreased, in abstracts that were accepted following a change in strategic focus to explicitly reduce weight bias and stigma. Additionally, the number of accepted abstracts that included a discussion of weight bias at the conferences increased over time. These findings offer some indication that the setting of strategic priorities to reduce weight bias and stigma can be helpful, although similar actions across multiple settings may be necessary to further shift the prevailing stigmatizing narratives that remain prevalent in society.
